# Diagnostic Delay Among Pulmonary Tuberculosis Patients Before, During and After COVID-19 Pandemic in Yichang City, China: A Longitudinal Study Based on Tuberculosis Surveillance Data

**DOI:** 10.1007/s44197-025-00419-5

**Published:** 2025-05-26

**Authors:** Jiamei Shao, Hao Zhang, Ye Wang, Xiaoyou Su, Hualei Xin, Ping Zhou, Zhili Li, Lei Wang, Jianxing Yu, Jianhua Liu, Zhongjie Li

**Affiliations:** 1 School of Population Medicine and Public Health, Chinese Academy of Medical Sciences & Peking Union Medical College, Beijing, China; 2 Yichang Center for Diseases Control and Prevention, Yichang, China; 3 Beijing Chest Hospital, Capital Medical University/Beijing Tuberculosis and Thoracic Tumor Research Institute, Beijing, China

**Keywords:** Pulmonary tuberculosis, Diagnostic delay, Longitudinal study

## Abstract

**Objectives:**

Early diagnosis of pulmonary tuberculosis (PTB) is essential for individual case treatment and community transmission control. However, the impact of the COVID-19 pandemic on PTB diagnosis remains inadequately understood. In this study, we aimed to investigate the diagnostic delay in patients with PTB before, during and after the COVID-19 pandemic.

**Methods:**

We conducted a longitudinal study of PTB in Yichang City from 2005 to 2023, utilizing data from the Tuberculosis Information Management System of China. The distribution of diagnostic delay (DD) was analyzed across three periods: pre-pandemic, during the pandemic, and post-pandemic. Multivariate mixed-effects logistic regression models were employed to identify factors associated with prolonged DD, defined as a delay exceeding 28 days.

**Result:**

A total of 58,774 patients with PTB were included in this study. The average annual number of cases was 3,293 pre-pandemic, 2,319 during the pandemic, and 2,426 post-pandemic. The fitted median DD in the pre-pandemic period (31.7 days, interquartile range [IQR] = 13.8–72.8) was significantly longer than that in the pandemic period (23.8 days, IQR = 11.3–50.3) and the post-pandemic period (20.6 days, IQR = 9-47.1) (*p* < 0.01). Elder patients aged 65 years and older had a longer median DD (32 days, IQR = 14.2–72.0) than patients aged 18–64 years (median: 30.1 days, IQR = 13.1–68.9) and patients under 18 years (median: 19.5 days, IQR = 8.6–44.2) (*p* < 0.01). Patients residing in rural areas also had a longer median DD (31 days, IQR = 14.2–72.0) compared to those in urban (median: 29.4 days, IQR = 13.7–70.2) (*p* < 0.01). Older age (adjusted Odds Ratio [aOR] = 2.20, 95% confidence interval [95% CI] = 2.00-2.42), rural residence (aOR = 1.10, 95% CI 1.06–1.14), positive pathogen testing (aOR = 1.35, 95% CI 1.23–1.49), and retreatment status (aOR = 1.23, 95% CI 1.16–1.31) were significantly associated with prolonged DD. Diagnosed by Xpert MTB/RIF (aOR = 0.71, 95% CI 0.65–0.78) was associated with a shorter DD.

**Conclusions:**

Compared to the pre-pandemic period, the overall interval from the onset of symptoms to the diagnosis of PTB patients shortened during and post-COVID-19 pandemic. Additional improvements in early diagnosis are needed for elderly patients and rural residents through the use of reliable diagnostic methods.

**Supplementary Information:**

The online version contains supplementary material available at 10.1007/s44197-025-00419-5.

## Introduction

Tuberculosis remains one of the leading infectious diseases threatening global public health. In 2023, there were an estimated 10.8 million reported cases and 1.25 million deaths worldwide due to tuberculosis [1]. In China, despite significant progress in tuberculosis control over the past decade, it estimated that approximately 734,000 new tuberculosis cases occurred according to the World Health Organization’s Global Tuberculosis Report 2024.

Pulmonary tuberculosis (PTB) is bacteriologically confirmed or clinically diagnosed tuberculosis in the lung parenchyma or the tracheobronchial tree which is primarily transmitted through the respiratory tract, with the general population being susceptible [2]. To reduce the global burden of tuberculosis, the World Health Organization (WHO) recommends prioritizing diagnostic interventions to improve early detection [3]. However, tuberculosis is often asymptomatic or presents with non-specific symptoms in the early stages. In most countries, passive case detection is the primary method for identifying tuberculosis cases, which means that by the time patients first seek care at health facilities, the disease may have already progressed for several weeks [4].

Timely diagnosis of PTB is essential for both individual treatment and the control of community transmission [5]. Indicators measuring the timeliness of PTB diagnosis include delays in consultation, delays in the health system, and delays in diagnosis [3]. In many high-burden countries, the implementation of new, reliable diagnostic technologies and active PTB screening strategies for high-risk groups has improved the timeliness of PTB patient detection [6]. However, the COVID-19 pandemic, which began in 2020, significantly impacted global health service systems, including case detection and management for diseases like PTB. These effects were unevenly distributed across different countries and regions [1]. In China, a strict zero COVID-19 policy was enforced nationwide from 2020 to 2022, but the impact of this policy on the timeliness of PTB diagnosis remains poorly understood [7]. A clearer understanding of how COVID-19-related restrictions and diagnostic improvements influenced delays in PTB will help predict how future pandemics may affect infectious diseases and guide more effective interventions and preparedness efforts [8].

In this study, we extracted PTB patient data of Yichang City (a medium-sized city in China) for the period 2005–2023 from the tuberculosis information management system in China. We compared changes of PTB diagnostic delays across three periods (pre-, during and post-COVID-19 pandemic), and explored the potential factors influencing these delays. This approach enhances further understanding of the impact of the COVID-19 pandemic and its response strategy impact on the PTB surveillance system, and will inform strategies to expedite the reduction of the PTB burden in the post-pandemic era.

## Methods

### Data Source

Yichang prefecture, a medium-sized city ocated in central China, is selected as the study setting, where had a resident population of 3,924,000 and a per capita GDP of $20,119 in 2023 year. PTB is classified as a Class-B infectious disease under the Law of the People’s Republic of China on the Prevention and Control of Infectious Diseases. A national tuberculosis information management system (TBIMS) has been established and is maintained by the Chinese Center for Disease Control and Prevention (China CDC) [9]. TBIMS is a population-based surveillance system in which all confirmed or clinically diagnosed tuberculosis cases are required to be reported. The system collects individual-level data, including demographic information (sex, age, occupation, living area), diagnosis type (clinically diagnosed or confirmed), date of symptom onset, date of diagnosis, diagnosis methods (sputum smear results, MTB culture results, Xpert MTB/RIF), and registration category, etc. (See Table S1 for detailed variable information). We extracted the complete records of individual PTB cases reported in TBIMS from January 1, 2005 to December 31, 2023.

### Case Definition

Our study included clinically and confirmed diagnosed PTB cases reported to the TBIMS, in accordance with the diagnostic criteria and case classification guidelines issued by the Chinese national health authorities [10] (Table S2). Clinically diagnosed cases were identified based on chest imaging, supplemented by clinical manifestations, immunology tests (e.g. tuberculin skin test, interferon gamma release assay, MTB antibody test), pathological examination or bronchoscopy. Confirmed cases were diagnosed based on a comprehensive assessment involving chest imaging, bacteriological tests, molecular biological tests, and lung tissue pathological examination [10].

We defined a new PTB case as a patient who had never received treatment for PTB or had received anti-PTB treatment for less than one month. A retreatment case was defined as an individual who has previously received anti-tuberculosis treatment for more than one month, but whose treatment had failed, relapsed, or been interrupted, resulting in the recurrence of active pulmonary tuberculosis [11].

### Statistical Analysis

Our analysis included all clinically diagnosed and confirmed PTB cases reported in Yichang City between 2005 and 2023. Diagnostic delay (DD) was defined as the number of days between symptom onset and diagnosis [12]. We fitted the distribution of DD using Gamma, Log-normal, Exponential, and Weibull distributions, selecting the distribution with the lowest Akaike information criterion (AIC) score as the best fit. DD was categorized into three periods: pre-pandemic (from January 1, 2005 to December 31, 2019), during the COVID-19 pandemic (from January 1, 2020 to January 8, 2023), and post-pandemic (from January 9, 2023 to December 31, 2023). These periods were determined based on (i) the date China officially announced the COVID-19 outbreak (December 31, 2019) [13]; and (ii) the date when China lifted COVID-19 control measures (January 8, 2023) [14]. Patients were grouped into three age categories: children (≤ 18 years), adults (19–64 years), and the elderly (≥ 65 years). Living areas were classified as urban (patients residing in cities and districts) and rural (patients residing in counties and townships). In the mixed effects model, gender, occupation, AFB smear, MTB culture, Xpert MTB/RIF, living area, pathogen results and treatment category were included as unordered categorical variables. Age group and period were treated as ordinal categorical variables, with the smallest category used as the reference. Year was included as a quantitative variable.

We used Chi-square tests or Fisher’s exact tests to analyze categorical variables and Wilcoxon rank-sum or Kruskal–Wallis tests for continuous variables as appropriate. A two-sided P-value of < 0.05 was considered statistically significant. To analyze factors associated with DD, we calculated the frequency and proportions of PTB patients with prolonged DD (defined as DD > 28 days) [15, 16], based on the 2004 Tuberculosis Control and Assessment Protocol by the Chinese Ministry of Health. Factors associated with prolonged DD were examined using mixed-effects logistic regression models. To account for provider effects, diagnosing medical institutions were included as a random effect in the model [17]. Data were presented as adjusted odds ratio (aORs) with a 95% confidence interval (95% CI). All statistical analyses were performed using R (version 4.3.3).

## Results

### Characteristics of Patients with PTB

Between 2005 and 2023, a total of 65,520 cases with tuberculosis were reported in Yichang City. We excluded 1,346 patients with tuberculous pleurisy or other extrapulmonary tuberculosis. An additional 5,400 patients were excluded due to missing data on the date of illness onset or diagnosis. In total, 58,774 (89.7%) PTB cases were included in the analysis. The annual PTB incidence ranged from 60.02 per 100 000 population in 2021 to 100.22 per 100 000 population in 2008, with a mean incidence of 78.07 per 100 000 population (Table S3). Among the included cases, 40,968 (69.7%) were male, and 34,613 (58.9%) lived in rural areas. The annual incidence of PTB patients declined from 4,017 cases in 2008 to 2,116 cases in 2023. Of the included patients, 27,447 (46.7%) were confirmed cases while 31,327 (53.3%) were clinically diagnosed cases.

The demographic and socioeconomic characteristics of PTB patients varied significantly across pre-pandemic, pandemic and post-pandemic periods. Notably, the median age of PTB during the pre-pandemic period (52 years) was lower than in the pandemic (59 years) and post-pandemic (63 years) periods (*p* < 0.01). A higher proportion of PTB patients lived in rural areas during the pandemic period compared to the pre- and post-pandemic periods (61.28% vs. 58.6% and 57.91%, respectively; *p* < 0.01). The proportion of farmers among PTB cases decreased from 67.79% in the pre-pandemic period to 54.53% in the post-pandemic period *(p* < 0.01). The proportion of PTB patients diagnosed as Xpert MTB/RIF positive increased markedly from 0.42% in the pre-pandemic period to 48.47% in the post-pandemic period (*p* < 0.01), while the percentage of culture-positive cases also increased from 3.91 to 32.44% over the same periods (Table 1).

**Table 1:** Characteristics of pulmonary tuberculosis patients in Yichang, stratified by study periods

Characteristics	Total (*n* = 58774)	Pre-pandemic^a^ (*n* = 49391)	During pandemic^b^ (*n* = 6957)	Post-pandemic^c^ (*n* = 2426)	*p*-value
Gender, no (%)					< 0.01
Male	40,968 (69.7)	34,575 (70.00)	4728 (69.76)	1665 (68.63)	
Female	17,806 (30.3)	14,816 (30.00)	2229 (32.04)	761 (31.37)	
Median age, years (IQR)	53 (38–65)	52 (36–64)	59 (49–69)	63 (52–72)	< 0.01
Age group, no (%)					< 0.01
≤ 18 years	2065 (3.5)	1846 (3.74)	174 (2.50)	45 (1.85)	
19–64 years	41,251 (70.2)	35,836 (72.56)	4184 (60.14)	1231 (50.74)	
65 + years	15,458 (26.3)	11,709 (23.71)	2599 (37.36)	1150 (47.40)	
Occupations, no (%)					< 0.01
farmers	38,846 (66.1)	33,484 (67.79)	4039 (58.06)	1323 (54.53)	
unemployment	10,060 (17.1)	7171 (14.52)	2093 (30.08)	796 (32.81)	
others	9868 (16.8)	8736 (17.7)	825 (11.9)	307 (12.7)	
DST, no (%)					< 0.01
Drug susceptible TB	57,210(97.4)	48,258 (97.71)	6637 (95.40)	2315 (95.42)	
Mono-resistance pattern	1153 (2.0)	824 (1.67)	248 (3.56)	81 (3.34)	
MDR-TB^d^	411(0.7)	309 (0.63)	72 (1.03)	30 (1.24)	
AFB smear, n (%)					< 0.01
Not detected^#^	35,086 (59.7)	29,395 (59.5)	4293 (61.7)	1398 (57.6)	
Positive	23,688 (40.3)	19,996 (40.5)	2664 (38.3)	1028 (42.37)	
MTB culture, n (%)					< 0.01
Not detected^#^	54,218 (92.2)	47,461 (96.09)	5118 (73.57)	1629 (67.56)	
Positive	4556 (7.8)	1930 (3.91)	1839 (26.43)	787 (32.44)	
Xpert MTB/RIF, n (%)					< 0.01
Not detected^#^	55,367 (94.2)	49,184 (99.58)	4933 (70.91)	1250 (51.53)	
Positive	3407 (5.8)	207 (0.42)	2024 (29.09)	1176 (48.47)	
Pathogen result, n (%)					< 0.01
Not detected^#^	31,327 (53.3)	27,743 (56.17)	2875 (41.33)	709 (29.23)	
Positive	27,447 (46.7)	21,648 (43.8)	4082 (58.67)	1717 (70.77)	
Living area, n (%)					< 0.01
Living in urban	24,161 (41.1)	20,446 (41.40)	2694 (38.72)	1021 (42.09)	
Living in rural	34,613 (58.9)	28,945 (58.60)	4263 (61.28)	1405 (57.91)	
Treatment category, n (%)					< 0.01
New case	53,633 (91.3)	45,154 (91.42)	6277 (90.23)	2202 (90.77)	
Retreatment case	4633 (7.9)	3773 (7.71)	651 (9.40)	209 (8.67)	
Missing	508 (0.8)	464 (0.94)	29 (0.42)	15 (0.62)	

a: from January 1, 2005 to December 31, 2019

b: from January 1, 2020 to January 8, 2023

c: from January 9, 2023 to December 31, 2023

d: Multidrug-Resistant Tuberculosis

^#^ Not detected: including negative testing result and the test not being conducted

### Diagnosis Delay

**Fig. 1:**
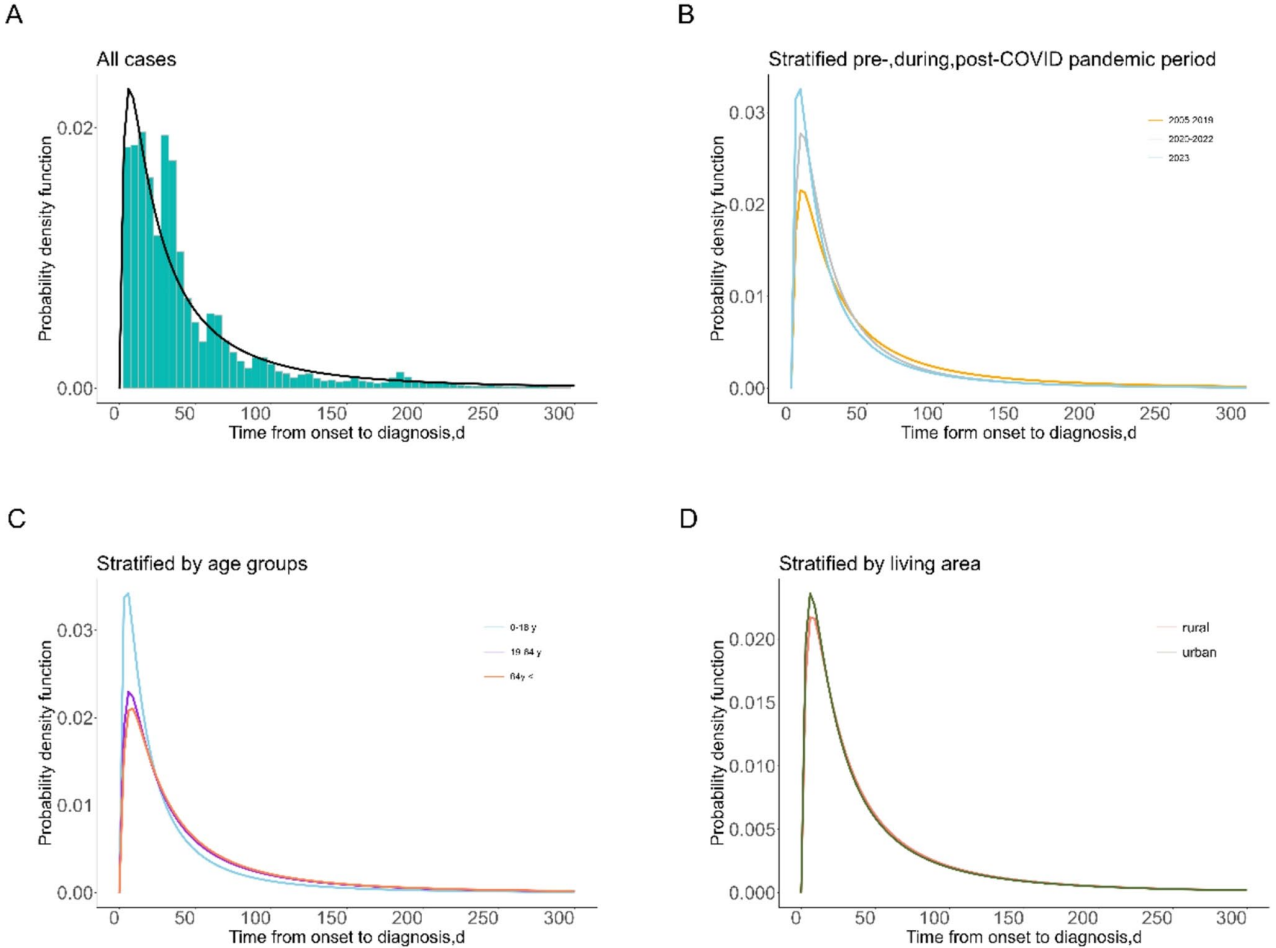
Estimated diagnosis delay days between symptom onset and diagnosis of PTB patients in Yichang. (**A**) For all case; (**B**) For different period; (**C**) For different age group; (**D**) For different living area

Among the 58,774 PTB patients, the actual median diagnostic delay (DD) was 31 days (IQR = 15–60). The median DD was 32 days (IQR: 15–62) in the pre-pandemic period, 27 days (IQR: 12–43) during the pandemic, and 22 days (IQR: 10–42) in the post-pandemic period. Based on the AIC criterion, the log-normal distribution provided the best fit for the DD data (AIC = 189176.3) (Table S4). The fitted median DD was estimated to be 30.1 days (95% Confidence Interval = 2.72-332.36) (Fig. 1, panel A). The median DD during the pre-pandemic period (31.7 days, IQR = 13.8–72.8) was significantly longer than that during the pandemic (23.8 days, IQR = 11.3–50.3) and post-pandemic period (20.6 days, IQR = 9-47.1) (*p < 0.01*). Elderly patients aged 65 years and above (32 days, IQR = 16–62) had longer DD compared to adults (31 days, IQR = 15–60) and children (22 days, IQR = 16–62) (*Pp < 0.01*). Patients living in rural areas (32 days, IQR = 15–61) had a longer median DD than those living in urban areas (31 days, IQR = 14–59) (*p < 0.01*) (Table 2). A significant decreasing trend in DD was observed from 2005 to 2023 (*p < 0.01*) (Figure S1).

**Table 2:** Actual diagnosis delay of pulmonary tuberculosis patients in Yichang, 2005–2023

Characteristics	Total, Median (IQR)	Pre-pandemic^a^ Median (IQR)	During pandemic^b^ Median (IQR)	Post-pandemic^c^ Median (IQR)	*p*-value
All patients	31 (15–60)	32 (15–62)	27 (12–43)	22 (10–42)	< 0.01
Gender					0.04
Male	31 (15–59)	32 (15–61)	26 (12–43)	23 (9–43)	
Female	31 (15–61)	32 (16–62)	27 (13–45)	22 (10–41)	
Age group					< 0.01
≤ 18 years	22 (16–62)	32 (15–62)	26 (12–43)	22 (9–40)	
19–64 years	31 (15–60)	23 (9–41)	18 (10–35)	11 (6–26)	
65 + years	32 (16–62)	33 (17–64)	28 (13–44)	24 (11–47)	
Occupations					< 0.01
farmers	31 (15–62)	32 (16–64)	27 (14–44)	27 (12–51)	
others	32 (15–53)	33 (17–58)	30 (12–45)	21 (8–36)	
AFB smear					< 0.01
Not detected^#^	31 (15–53)	31 (15–55)	29 (14–44)	26 (12–44)	
Positive	32 (14–66)	33 (15–70)	22 (9–41)	17 (6–40)	
MTB culture					< 0.01
Not detected^#^	31 (15–61)	32 (15–62)	26 (13–44)	24 (11–43)	
Positive	30 (12–46)	33 (18–52)	27 (10–42)	20 (7–41)	
Xpert MTB/RIF					< 0.01
Not detected^#^	31 (15–61)	32 (15–62)	28 (13–45)	25 (11–42)	
Positive	22 (9–41)	30 (9–39)	22 (10–40)	21 (8–42)	
Pathogen result					< 0.01
Not detected^#^	31 (15–53)	31 (15–55)	29 (15–46)	28 (14–43)	
Positive	31 (14–64)	33 (15–68)	23 (10–41)	20 (7–42)	
Living area					< 0.01
Living in rural	32 (15–61)	33 (16–63)	26 (14–42)	30 (14–56)	
Living in urban	31 (14–59)	31 (15–61)	27 (11–45)	18 (7–36)	
Treatment category					< 0.01
New case	31 (15–57)	31 (15–60)	26 (12–43)	22 (10–42)	
Retreatment case	34 (16–76)	35 (17–93)	28 (12–50)	25 (7–43)	

a: 2005–2019

b: 2020–2022

c: 2023

^#^ Not detected: including negative testing result and the test not being conducted.

### Risk Factor for Prolonged Diagnostic Delay of PTB

Univariable analysis revealed significant differences in the proportion of PTB cases with prolonged DD across study periods, age groups, living areas, diagnostic methods, and treatment categories (all *p* < 0.05), which were subsequently included in the multivariate analysis. Multivariate mixed-effects logistic regression analysis showed that both the year and diagnostic institutions were significantly associated with prolonged DD in PTB cases (*p < 0.01*). The risk of prolonged DD was increased by 1.11 times (95% CI = 1.07–1.15) each year from 2005 to 2023. After adjusting for diagnostic institutions and the yearly trend, the COVID-19 pandemic continued to impact the timeliness of PTB diagnosis. PTB cases during the COVID-19 pandemic period (aOR = 0.80, 95% CI = 0.75–0.86) and post-COVID-19 pandemic period (aOR = 0.63, 95% CI = 0.57–0.70) were less likely to experience prolonged DD compared to the pre-pandemic period. Other factors significantly associated with prolonged DD in the model included being elderly (65 + years) (aOR = 2.20, 95% CI = 2.00-2.42), positive pathogen testing results (aOR = 1.35, 95% CI 1.23–1.49), retreatment cases (aOR = 1.23, 95% CI = 1.16–1.31), and living in rural (aOR = 1.11, 95% CI = 1.07–1.15) were identified as risk factors significantly associated with prolonged DD (Table 3). Diagnosis by Xpert MTB/RIF (aOR = 0.71, 95% CI = 0.65–0.78) and AFB smear (aOR = 0.75, 95% CI = 0.69–0.82) were associated with shorter DD (Table 3). Sensitivity analyses stratified by period and age group showed similar results (Table S5, Table S6).

**Table 3:** Multivariate mixed-effects logistic regression analysis results of factors associated with prolonged diagnosis delay of PTB patients in Yichang City, 2005–2023

Characteristics	Total	DD > 28 days, no. (%)	Crude ORs (95% CI)	Adjusted ORs (95% CI)
Period				
2005–2019	49,391	27,931 (56.6)	reference	reference
2020–2022	6957	3304 (47.5)	0.73 (0.69–0.77)	0.80 (0.75–0.86)
2023	2426	1028 (42.4)	0.53 (0.49–0.58)	0.63 (0.57–0.70)
Year			0.84 (0.83–0.86)	1.11 (1.07–1.15)
Age group, years				
≤ 18	2065	852 (41.26)	reference	reference
19–64	41,251	22,547 (54.7)	1.72 (1.57–1.88)	1.81 (1.65–1.98)
65+	15,458	8864 (57.3)	1.91 (1.74–2.10)	2.2 (2.00-2.42)
Sex				
Male	40,968	22,337 (54.5)	reference	reference
female	17,806	9926 (55.8)	1.05 (1.01–1.08)	1.08 (1.04–1.11)
AFB smear				
Not detected^#^	35,086	19,043 (54.3)	reference	reference
Positive	23,688	13,220 (55.8)	1.06 (1.03–1.10)	0.75 (0.69–0.82)
MTB culture				
Not detected^#^	54,218	29,893 (55.1)	reference	reference
Positive	4556	2370 (52.0)	0.88 (0.83–0.94)	0.95 (0.88–1.03)
Xpert MTB/RIF				
Not detected^#^	55,367	30,808 (55.6)	reference	reference
Positive	3407	1455 (42.7)	0.59 (0.55–0.64)	0.71 (0.65–0.78)
Pathogen result				
Not detected^#^	31,327	17,029 (54.4)	reference	reference
Positive	27,447	15,234 (55.5)	1.05 (1.01–1.08)	1.35 (1.23–1.49)
Living Area				
Living in urban	24,161	18,691 (54.0)	reference	reference
Living in rural	34,613	13,572 (56.2)	1.09 (1.06–1.13)	1.10 (1.06–1.14)
Treatment category^*^				
New case	53,633	29,129 (54.3)	reference	reference
Retreatment case	4633	2748 (59.3)	1.23 (1.15–1.30)	1.23 (1.16–1.31)

Random effect: variance = 0.09 (*p* = 0.288)

^#^ Not detected: including negative testing result and the test not being conducted

^*^ Treatment category: In this part, the category “missing” was not contained, for it is meaningless

## Discussion

This longitudinal study analyzed the diagnostic delays of 58,774 PTB cases diagnosed in Yichang City from 2005 to 2023. We found that the improvements in the timeliness of PTB diagnosis during and after the COVID-19 pandemic were significant compared to the pre-pandemic period. Additionally, several factors were significantly associated with prolonged DD, including older age, living in rural areas, positive pathogen testing results, passive case finding, and retreatment. In contrast, diagnosed by Xpert MTB/RIF was associated with shorter DD.

In China, the management of PTB across all levels of medical facilities has been strengthened since 2005. In 2018, the responsibilities for PTB management was transferred to tuberculosis-specialized hospitals, which actively implemented proactive screening and launched the tuberculosis-free community initiative. As a result of these policies, the incidence of PTB in Yichang City has decreased steadily, and the diagnostic timeliness for PTB patients has improved year by year. The efforts have had a positive impact on local tuberculosis control and prevention [18–20]. Similar results were observed in a study conducted in Shanghai city in China from 2018 to 2020 [21], where a significant reduction in DD was noted following the COVID-19 pandemic. Despite the pandemic slightly affecting patient care, such as a notable decline in outpatient visits starting in 2020, overall diagnostic timeliness continued to improve [22]. In Zhu’s study, by August 2021, outpatient visits had increased by over 22.2% compared to the pre-pandemic level [23], indicating a significant increase in the public health awareness, enhanced sensitivity to respiratory symptoms (e.g., coughing) among general population and healthcare workers, and the increasing application of timely and accurate PTB testing methods in health facilities. During and after the COVID-19 pandemic, the use of Xpert MTB/RIF in Yichang increased nearly 100-fold compared to the pre-pandemic period. The use of rapid, sensitive and accurate laboratory diagnostics, such as Xpert MTB/RIF, contributed to improvement of PTB diagnosis during and after the COVID-19 pandemic in the region.

However, it is important to note that the median diagnostic delay for PTB in Yichang City was 22 days in 2023, which still needs to be shortened. More in-depth research is needed to identify factors that significantly influence diagnostic delay in the future.

Previous studies have found that age is an important factor significantly affecting the timeliness of PTB diagnosis, and older individuals generally experiencing longer DD. For example, Liu et al. found that the elderly population had longer DD for PTB [7, 24]. Consistent with these findings, our study revealed that elderly patients in Yichang City had a higher risk of prolonged DD. Additionally, the proportion of elderly PTB patients increased over the three study periods, demonstrating a year-by-year upward trend. The elderly people had longer diagnostic delays compared to the adult and adolescent, which may be due to their reduced capacity to access and utilize healthcare services, as well as transportation difficulties that further hinder timely diagnostic [25]. With China’s aging population project to reach 30% aged 60 years or older by 2035 [26], the challenges posed by population aging to PTB prevention and control are becoming increasingly apparent. Our findings highlight the urgent need for local health authorities to prioritize the elderly by enhancing awareness of PTB symptoms, strengthening health education, and encouraging active participation in screening programs, which are crucial for improving patient-centered PTB care.

Urban/rural areas is another important factor influencing the timeliness of PTB diagnosis. Zhu et al. found that individuals living in rural areas had longer PTB diagnostic delays [27, 28]. In our study, we observed poorer improvement in diagnostic delays among rural residents. One possible reason is the so-called “health migrant effect”, whereby healthier individuals with better access to healthcare tend to migrate from rural to urban areas, reflecting broader urbanization trends [29]. Another contributing factor is the better development and accessibility of healthcare resources, which increases health-seeking behavior and reduces diagnostic delays [30]. China’s urbanization has progressed rapidly, with the urbanization rate increasing from 42.99% in 2005 to 66.16% in 2023. In Yichang, the proportion of urban PTB cases rose from 51.25% to 62.57% over the same period. In our study, over half of PTB patients were farmers. Although the proportion of farmers among PTB cases decreased from 67.79% before the pandemic to 54.53% after, this group still warrants close attention. Our findings highlight the need enhance public health efforts targeting farmers in rural areas to improve PTB prevention and control. Local health authorities should strengthen the diagnostic and screening initiatives in these high-priority regions and among high-risk populations [31].

This study has several limitations. First, as a retrospective analysis, it relies on data extracted from the tuberculosis information management system, which may have inherent limitations such as missing data or recall bias. These factors may impact the exploration of the determinants of diagnostic delays. Second, our study focused solely on patients with pulmonary tuberculosis, so these findings cannot be generalized to patients with extrapulmonary tuberculosis, which warrants further investigation.

The strength of this study lies in its inclusion of all 58,774 PTB patients over a 19-year period. The large sample size and extended duration provide substantial statistical power to identify a wide range of factors associated with prolonged diagnostic delays (DD). We examined the distribution of diagnostic delays across different subgroups and their influencing factors, and explored the impact of the COVID-19 pandemic on PTB diagnostic patterns, an area less explored in previous studies.

## Conclusion

Compared to the pre-pandemic period, the overall interval from symptom onset to diagnosis among pulmonary tuberculosis patients declined gradually during the pandemic and post-pandemic period. However, the extent of this reduction varied across different subgroups. The timeliness of PTB diagnosis among the elderly and the residences in rural area requires further improvement with more reliable diagnostic approach.

## Electronic supplementary material

Below is the link to the electronic supplementary material.

Supplementary Material 1

## Data Availability

The datasets generated and/or analysed during the current study are not publicly available as TB Management Information System (TBIMS) is not an open access but an internal and administrative database for TB management in China, but are available from the corresponding author on reasonable request.
