# Supplementary material for: Diagnostic Delay Among Pulmonary Tuberculosis Patients Before, During and After COVID-19 Pandemic in Yichang City, China: A Longitudinal Study Based on Tuberculosis Surveillance Data
Source: J Epidemiol Glob Health. 2025 May 26;15(1):74. doi: 10.1007/s44197-025-00419-5 (PMC12106177; doi:10.1007/s44197-025-00419-5)

Supplementary Appendix

**Title:** Diagnosis Delay among Tuberculosis Patients Before, During and After COVID-19 Pandemic: A Longitudinal Study in Yichang City, China, 2005-2023

**Tables & Figures**

- **Table S1.** Major variables reported in the Tuberculosis Information Management System (TBIMS) and used in the study in Yichang City, 2005-2023
- **Table S2** Case definitions of tuberculosis in China
- **Table S3.** Incidence of pulmonary tuberculosis in Yichang City, 2005-2023
- **Table S4.** Model parameters of diagnostic delay days fitted to four different distributions
- **Table S5.** Analysis of factors influencing prolonged delay in tuberculosis diagnosis stratified by period, Yichang City, 2005-2023
- **Table S6.** Analysis of factor influencing prolonged delay in tuberculosis diagnosis stratified by age group, Yichang City, 2005-2023
- **Figure S1.** The distribution of the interval from symptom to diagnosis by different groups: all patient by year(A), age group(B), living area group(C)

**Table S1**. Major variables reported in the Tuberculosis Information Management System (TBIMS) and used in the study in Yichang City, 2005-2023

| Variables | Definition/Classification |
| --- | --- |
| Sex | Male and female |
| Age | Interval from the date of birth to onset date of illness |
| Zone code of residence address | Unique 8-digital number at county and township level |
| Occupation | Occupation of case patients |
| Type of diagnosis | Confirmed case |
|  | Clinical case |
| Date of illness onset | Date of illness onset |
| Date of diagnosis | Date of diagnosis as a probable or confirmed case |
| Date of report | First date of reporting to surveillance system |
| Diagnosis way |  |
|  | Pathogen negative |
|  | Acid-Fast Bacillus Smear(AFB smear) |
|  | Mycobacterium Tuberculosis Culture(MTB culture) |
|  | Xpert MTB/RIF |
| Registration category |  |
|  | New patient |
|  | Retreatment patient |

**Table S2.** Case definitions of tuberculosis in China

| **Type of diagnosis** | **Case Definitions** |
| --- | --- |
| Clinically diagnosed case | 1. Adults |
|  | 1）Chest image with lesions consistent with active tuberculosis |
|  | 2）Accompanied by any of the following |
|  | suspicious symptoms of tuberculosis. Tuberculin test moderately positive or more Positive γ-interferon release test Positive tuberculosis antibody test Extrapulmonary histopathology for tuberculosis Bronchoscopy consistent with tuberculosis |
|  | 2. Children |
|  | 1) Lesions consistent with active tuberculosis on chest imaging 2) With suspicious symptoms of tuberculosis 3) with a moderately positive tuberculin test or a positive gamma-interferon release test. |
| Laboratory-confirmed case | 1. Lung tissue cases consistent with pathological changes in tuberculosis |
|  | 2. Chest imaging with lesions consistent with active tuberculosis and one of the following conditions is met: |
|  | 1) Positive nucleic acid test for Mycobacterium tuberculosis 2) Positive culture of Mycobacterium tuberculosis 3) One sputum smear acid-fast bacillus test positive.; |
|  | 3. Two sputum smears acid-fast bacillus test positive |
|  | 4. One sputum smear acid-fast bacillus test positive and one sputum culture positive for Mycobacterium tuberculosis |

**Table S3.** Incidence of pulmonary tuberculosis in Yichang City, 2005-2023

| year | Incidence number | Incidence rate* |
| --- | --- | --- |
| 2005 | 2604 | 65.299 |
| 2006 | 3300 | 82.453 |
| 2007 | 3274 | 81.645 |
| 2008 | 4017 | 100.217 |
| 2009 | 3597 | 89.618 |
| 2010 | 3449 | 86.539 |
| 2011 | 3489 | 87.479 |
| 2012 | 3451 | 86.498 |
| 2013 | 3425 | 85.608 |
| 2014 | 3186 | 79.570 |
| 2015 | 3137 | 78.783 |
| 2016 | 2998 | 76.032 |
| 2017 | 3108 | 79.233 |
| 2018 | 3152 | 80.435 |
| 2019 | 3204 | 81.956 |
| 2020 | 2513 | 64.452 |
| 2021 | 2328 | 60.017 |
| 2022 | 2116 | 54.742 |
| 2023 | 2426 | 62.762 |
| *:Incidence rate based on per 100,000 population |  |  |

**Table S4.** Model parameters of diagnostic delay days fitted to four different distributions

| Parametric model |  | Diagnosis interval (days) | | |
| --- | --- | --- | --- | --- |
|  |  | Median | 95%CI | AIC |
| Gamma |  | 30.99 | 3.17-117.47 | 625448.3 |
| Lognormal |  | 30.10 | 2.72-332.36 | 189176.3 |
| exponential |  | 62.17 | 2.27-330.84 | 640949.1 |
| Weibull |  | 33.10 | 0.26-383.99 | 608424.3 |

CI: 95% Confidence Interval; AIC: Akaike Information Criterion (a lower AIC indicates a better model fit).

**Table S5.** Analysis of factors influencing the long delay in tuberculosis diagnosis stratified by period, Yichang City, 2005-2023

| **Characteristics** | **Pre-pandemic** | **During pandemic** | **Post-pandemic** |
| --- | --- | --- | --- |
| Year | 1.13(1.09-1.17) | 0.74(0.54-1.02) | —— |
| Age group, years |  |  |  |
| ≤18 | reference | reference | reference |
| 19-64 | 1.82(1.65-2.00) | 1.66(1.21-2.28) | 2.99(1.43-6.25) |
| 65+ | 2.25(2.03-2.49) | 1.92(1.39-2.65) | 3.64(1.74-7.62) |
| Sex |  |  |  |
| Male | reference | reference | reference |
| female | 1.09(1.05-1.13) | 1.06(0.95-1.17) | 0.89(0.74-1.06) |
| AFB smear |  |  |  |
| Not detected^#^ | reference | reference | reference |
| Positive | 0.69(0.59-0.8) | 0.79(0.69-0.91) | 0.74(0.59-0.91) |
| MTB culture |  |  |  |
| Not detected^#^ | reference | reference | reference |
| Positive | 0.92(0.79-1.06) | 1.19(1.03-1.37) | 0.97(0.79-1.19) |
| Xpert MTB/RIF |  |  |  |
| Not detected^#^ | reference | reference | reference |
| Positive | 0.7(0.52-0.93) | 0.88(0.76-1.004) | 1.01(0.81-1.27) |
| Pathogen result |  |  |  |
| Not detected^#^ | reference | reference | reference |
| Positive | 1.55(1.32-1.82) | 0.87(0.72-1.05) | 0.74(0.54-1.004) |
| Living Area |  |  |  |
| Living in urban | reference | reference | reference |
| Living in rural | 1.08(1.04-1.13) | 1.07(0.95-1.2) | 1.66(1.35-2.05) |
| Treatment category^*^ |  |  |  |
| New case | reference | reference | reference |
| Retreatment case | 1.25(1.17-1.34) | 1.06(0.9-1.24) | 1.17(0.87-1.57) |
| Not detected: including negative testing result and the test not being conducted. * Treatment category: In this part, the category “missing” was not contained, for it is meaningless. "——"：There is only one year during the post-pandemic period. | | | |

**Table S6.** Analysis of factor influencing prolonged delay in tuberculosis diagnosis stratified by age group, Yichang City, 2005-2023

| **Characteristics** | **age≦18** | **18<age<65** | **age≧65** |
| --- | --- | --- | --- |
| period |  |  |  |
| Pre-pandemic | reference | reference | reference |
| During pandemic | 0.84(0.59-1.19) | 0.85(0.80-0.92) | 0.85(0.78-0.93) |
| Post-pandemic | 0.41(0.20-0.87) | 0.85(0.80-0.92) | 0.72(0.64-0.82) |

**Figure S1.** The distribution of the interval from symptom to diagnosis by different groups: all patient by year(A), age group(B),living area group(C)


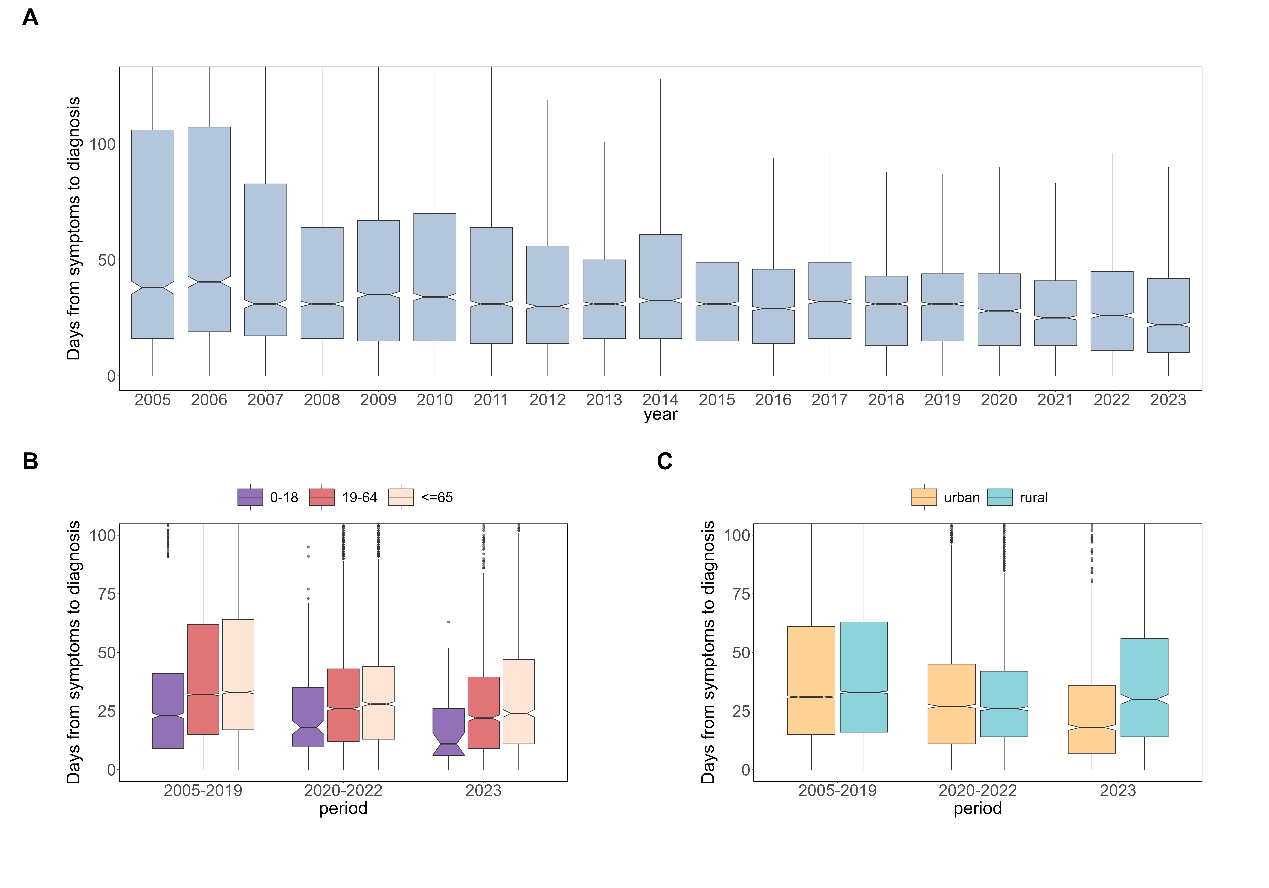

Supplement: Supplementary file 1 — Supplementary Material 1 [file 44197_2025_419_MOESM1_ESM.docx]
